# Risky and rushed public crack cocaine smoking: the potential for supervised inhalation facilities

**DOI:** 10.1186/s12889-016-3137-3

**Published:** 2016-06-07

**Authors:** Pauline Voon, Lianping Ti, Huiru Dong, M-J Milloy, Evan Wood, Thomas Kerr, Kanna Hayashi

**Affiliations:** Urban Health Research Initiative, British Columbia Centre for Excellence in HIV/AIDS, St. Paul’s Hospital, 608-1080 Burrard Street, Vancouver, BC V6Z 1Y6 Canada; School of Population and Public Health, Faculty of Medicine, University of British Columbia, 2206 East Mall, Vancouver, BC V6Z 1Z3 Canada; Department of Medicine, University of British Columbia, St. Paul’s Hospital, 608-1081 Burrard Street, Vancouver, BC V6Z 1Y6 Canada

**Keywords:** Crack, Cocaine, Public drug use, Rushed drug use, Supervised inhalation, Supervised consumption, Crack pipe distribution, Intravenous, Injection, Harm reduction

## Abstract

**Background:**

Despite the multitude of public health and community harms associated with crack cocaine use, little is known about factors associated with smoking crack in public and related risks such as rushed public crack smoking.

**Methods:**

Data were derived from two prospective cohort studies of people who use illicit drugs in Vancouver, Canada between 2010 and 2014. Multivariable generalized estimating equations were used to identify the prevalence and correlates of public crack smoking and rushed public crack smoking.

**Results:**

In total, 1085 participants who had smoked crack in the prior six months were eligible for the analysis, of which 379 (34.9 %) reported always or usually smoking crack in public in the previous six months at some point during the study period. Factors positively and independently associated with public crack smoking included public injection drug use (adjusted odds ratio [AOR]: 5.42, 95 % confidence interval [CI]: 3.76-7.82), homelessness (AOR: 3.48, 95 % CI: 2.77-4.36), at least daily crack use (AOR: 2.69, 95 % CI: 2.19-3.31), crack pipe sharing (AOR: 1.98, 95 % CI: 1.60-2.46), drug dealing (AOR: 1.59, 95 % CI: 1.30-1.94), recent incarceration (AOR: 1.47, 95 % CI: 1.09-1.98), noticing police presence when buying or using drugs (AOR: 1.30, 95 % CI: 1.06-1.60), and younger age (AOR: 1.03, 95 % CI: 1.01-1.04). Rushed public crack smoking, which was reported by 216 (28.8 %) of 751 participants who had smoked crack in public at least once during the study period, was positively and independently associated with homelessness (AOR: 2.61, 95 % CI: 1.96-3.49), at least daily crack use (AOR: 1.48, 95 % CI: 1.11-1.98), crack pipe sharing (AOR: 1.44, 95 % CI: 1.10-1.89), drug dealing (AOR: 1.39, 95 % CI: 1.04-1.86), and younger age (AOR: 1.02, 95 % CI: 1.01-1.04).

**Conclusions:**

A high prevalence of public crack smoking and rushed public crack smoking was observed in this setting. These findings point to the need for implementing and evaluating evidence-based public health interventions, such as supervised inhalation facilities, to reduce the risks and harms associated with smoking crack in public.

## Background

In recent years, increases in crack cocaine use in many North American settings have contributed to a “neglected epidemic” in which crack cocaine has remained among the most prevalent and easily obtainable illicit drugs [1–3]. National population-level surveys estimate that past-year crack cocaine use is prevalent in approximately 1.1 % and 0.3 % of the general adult populations in Canada and the United States, respectively [4, 5]. A recent multicriteria decision analysis performed by the Independent Scientific Committee on Drugs found that crack cocaine (herein referred to as “crack”) is among the top three illicit drugs that are most harmful to individual users and to others, due to its potential for causing substantial physical, psychological, and social harms [6]. Specifically, individuals who use crack have been found to have elevated risk of HIV, hepatitis C virus (HCV), tuberculosis, herpes zoster, and other infectious pathogens secondary to sores, burns, or cuts, either from shared crack pipes or from increased prevalence of sexual risk behaviors [7–14]. Additionally, crack use has been found to be associated with polysubstance use, comorbid mental illness, incarceration, and other health and social-structural problems, yet research indicates that individuals who use crack may be less likely to access health and social services [3, 15–17].

Crack is often smoked in public settings, which may contribute to public health risks such as hazardous debris from crack smoking paraphernalia potentially contaminated with infectious pathogens (e.g., from glass crack pipes), as well as crack-related street disorder such as public intoxication, dealing drugs in public, or related violence [18–20]. To date, much of the literature on public drug use has focused on the use of injection drugs in public and associated risks. Specifically, public injecting has been found to be associated with greater odds of injection-related risk behaviours (e.g., sharing used injection equipment, not cooking or filtering drugs prior to injecting), HIV and HCV transmission, and poor health and social status (e.g., more severe drug dependency, social isolation, and unstable lifestyles) [21–23]. However, little is known about risks associated with the use of non-injection drugs in public settings—particularly crack.

Furthermore, there is a dearth of research on factors associated with the use of non-injection drugs while rushed. Here again, much of the literature has concentrated on rushed injection drug use, which is often prompted by fear of police arrest or street violence, and which frequently involves risky injection behaviors (e.g., jabbing at veins, not testing the strength of drugs before injecting, re-using injecting equipment) that may lead to vein trauma, abscesses, overdose, bacterial infections, or infectious disease transmission [24–26]. The risks associated with rushed crack smoking are not well described, apart from one study which found a high prevalence of burns and inhaled metallic crack pipe filter screens among individuals who reported rushed crack smoking [27]. Therefore, this study sought to investigate the prevalence and correlates of public crack smoking and rushed public crack smoking among participants of two prospective cohorts of people who use illicit drugs in Vancouver, Canada.

## Methods

Data for these analyses were derived from two prospective observational cohorts in Vancouver, Canada: the AIDS Care Cohort to evaluate Exposure to Survival Services (ACCESS) of HIV-seropositive illicit drug users and the Vancouver Injection Drug Users Study (VIDUS) of HIV-seronegative injection drug users. These cohorts have previously been described in detail [28, 29]. In short, since 1996, more than 2000 individuals have been recruited into these cohorts through snowball sampling and street outreach methods in Vancouver’s Downtown Eastside, a post-industrial neighbourhood with an established drug market and widespread illicit drug use, poverty, poor housing conditions, and infectious diseases such as HIV and HCV [30].

Participants are eligible for VIDUS if they are HIV-seronegative, over 18 years of age, and have injected an illicit drug in the month prior to the baseline interview. Participants are eligible for ACCESS if they are HIV-seropositive, over the age of 18, and have used an illicit drug other than or in addition to cannabis within the month prior to the baseline interview. As these two cohorts were originally one single cohort until they were split into the two present cohorts in 2005, the two cohorts employ harmonized data collection and follow-up procedures to allow for combined analyses of the HIV-seronegative and HIV-seropositive study participants. Specifically, at baseline and semi-annually, participants answer an interviewer-administered questionnaire, which elicits data on demographic characteristics, drug-using behaviours and related exposures; provide blood samples for HIV (for VIDUS participants only) and HCV serologic analyses; and are referred as necessary to medical care and drug and alcohol treatment. All participants provide written informed consent and receive a $30 stipend at the end of each study visit. These studies have received annual ethics approval from the University of British Columbia and Providence Health Care Research Ethics Board.

The present analyses were restricted to interviews that were conducted between December 1, 2010 to May 31, 2014. Participants were eligible for this analysis if they reported smoking crack at least once in the six-month period prior to their interview, and if they had a history of ever injecting any drug at the time of interview. The main outcome measure of interest was smoking crack in public (always or usually vs. sometimes, occasionally or never) [31]. The self-reported demographic, behavioural, social and structural explanatory characteristics considered in the analyses were: age (per one-year decrease); gender (female vs. male); HIV serostatus (positive vs. negative); homelessness (yes vs. no); drug dealing (yes vs. no); sex work (yes vs. no); crack smoking (≥ daily vs. < daily); sharing a crack pipe (yes vs. no); binge non-injection drug use (yes vs. no); heroin injection (≥ daily vs. < daily); cocaine injection (≥ daily vs. < daily); crystal methamphetamine injection (≥ daily vs. < daily); public injection drug use (always or usually vs. sometimes, occasionally or never); noticing police presence when buying or using drugs (yes vs. no); being stopped, searched or detained without arrest by police (yes vs. no); recent incarceration (yes vs. no); enrolment in drug addiction treatment, excluding methadone (yes vs. no); and being a victim of violence (yes vs. no). All variables referred to activities or events in the six months prior to the participant’s interview, unless otherwise indicated. As in previous studies, binge non-injection drug use was defined as any period of time within the previous six months from the time of interview during which any drugs were used more frequently than usual [32].

First, we compared those who did and did not smoke crack in public at baseline using Pearson’s Chi-squared test for dichotomous variables and the Wilcoxon rank sum test for continuous variables. Next, since analyses of factors potentially associated with public crack smoking included serial measures for each subject, we used generalized estimating equations (GEE) with logit link function and exchangeable working correlation structure to account for correlations between repeated measurements. Bivariable GEE analyses were conducted to obtain unadjusted odds ratios and *p*-values for factors associated with public crack smoking. Using an *a priori*-defined statistical protocol based on examination of the quasi-likelihood under the independence model criterion (QIC) value, a preliminary multivariable model was built using all variables that were significantly associated with the outcome at the *p* < 0.10 in the bivariable analyses. Next, each variable with the highest *p*-value was removed sequentially. The final model included the set of variables associated with the lowest QIC, and was assessed with variance inflation factors to ensure the absence of multicollinearity.

As a secondary analysis, among participants who reported any public crack smoking (i.e., always, usually, sometimes, or occasionally), bivariable and multivariable GEE analyses were conducted to determine factors associated with rushed public crack smoking, using the same procedures detailed above. All *p*-values were two sided. A significant association was defined as *p* < 0.05. All statistical analyses were performed using the SAS software version 9.4 (SAS, Cary, NC).

## Results

Of the 1085 participants eligible for the present analysis, 400 (36.9 %) were female. The median age at the first study visit was 46 (interquartile range: 40—52) years. Participants in this sample contributed to a total of 5126 observations during the study period. Table 1 shows the baseline sample characteristics. At the first study visit, 244 (22.5 %) participants reported “always” or “usually” smoking crack in public in the previous six months. During the 42-month study period, 379 (34.9 %) participants reported “always” or “usually” smoking crack in public at least once.

**Table 1:** Baseline characteristics of VIDUS and ACCESS participants who smoked crack in Vancouver, Canada, stratified by public crack smoking (*n* = 1085)

Characteristic	Smoked crack in public^a^244 (22.5 %) n (%)	Did not smoke crack in public^b^841 (77.5 %) n (%)	Odds ratio (95 % CI)	*p -* value
Age				
Median years	42.1	47.2	1.05 (1.04, 1.08)	<0.001
(IQR)	(36.1 – 47.0)	(40.7 – 52.5)		
Gender				
Female	93 (38.1)	307 (36.5)	1.07 (0.80, 1.44)	0.646
Male	151 (61.9)	534 (63.5)		
HIV serostatus				
Positive	90 (36.9)	406 (48.3)	0.63 (0.47, 0.84)	0.002
Negative	154 (63.1)	435 (51.7)		
Homelessness^c,d^				
Yes	115 (47.1)	109 (13.0)	6.02 (4.36, 8.31)	<0.001
No	128 (52.5)	730 (86.8)		
Drug Dealing^c^				
Yes	106 (43.4)	156 (18.5)	3.37 (2.48, 4.59)	<0.001
No	138 (56.6)	685 (81.5)		
Sex work^c, a^				
Yes	40 (16.4)	95 (11.3)	1.56 (1.05, 2.33)	0.029
No	201 (82.4)	745 (88.6)		
Crack smoking^c,d^				
≥ Daily	136 (55.7)	254 (30.2)	2.90 (2.16, 3.89)	<0.001
< Daily	108 (44.3)	585 (69.6)		
Shared crack pipe^c^				
Yes	143 (58.6)	384 (45.7)	1.68 (1.26, 2.25)	<0.001
No	101 (41.4)	457 (54.3)		
Binge non-injection drug use^c^				
Yes	99 (40.6)	341 (40.5)	1.01 (0.75, 1.34)	0.994
No	145 (59.4)	500 (59.5)		
Heroin injection^c^				
≥ Daily	78 (32.0)	113 (13.4)	3.03 (2.17, 4.23)	<0.001
< Daily	166 (68.0)	728 (86.6)		
Cocaine injection^c,d^				
≥ Daily	28 (11.5)	43 (5.1)	2.40 (1.46, 3.95)	<0.001
< Daily	216 (88.5)	796 (94.6)		
Crystal meth injection^c^				
≥ Daily	10 (4.1)	27 (3.2)	1.29 (0.61, 2.70)	0.501
< Daily	234 (95.9)	814 (96.8)		
Public injection drug use^c,d^				
Always or usually	59 (24.2)	19 (2.3)	13.80 (8.04, 23.72)	<0.001
Sometimes, occasionally or never	184 (75.4)	818 (97.3)		
Noticed police presence^c,d^				
Yes	175 (71.7)	536 (63.7)	1.47 (1.07, 2.02)	0.016
No	67 (27.5)	302 (35.9)		
Stopped, searched or detained without arrest by police^c,d^				
Yes	57 (23.4)	90 (10.7)	2.62 (1.81, 3.80)	<0.001
No	181 (74.2)	750 (89.2)		
Incarceration^c, a^				
Yes	43 (17.6)	55 (6.5)	3.13 (2.04, 4.81)	<0.001
No	196 (80.3)	785 (93.3)		
Drug addiction treatment (excluding methadone)^c,d^				
Yes	148 (60.7)	525 (62.4)	0.96 (0.71, 1.28)	0.766
No	92 (37.7)	312 (37.1)		
Victim of violence^c,d^				
Yes	39 (16.0)	128 (15.2)	1.08 (0.73, 1.59)	0.710
No	200 (82.0)	707 (84.1)		

^a^Defined as “always” or “usually” smoking crack in public in the previous six months

^b^Defined as “sometimes,” “occasionally” or “never” smoking crack in public in the previous six months

^c^Denotes activities/events in the previous six months

^d^Counts may not add up to column totals due to missing responses

Table 2 presents the results of the bivariable and multivariable GEE analyses. In multivariable GEE analyses, factors that remained positively and independently associated with public crack smoking included: public injection drug use (adjusted odds ratio [AOR]: 5.42, 95 % confidence interval [CI]: 3.76-7.82), homelessness (AOR: 3.48, 95 % CI: 2.77-4.36), at least daily crack use (AOR: 2.69, 95 % CI: 2.19-3.31), crack pipe sharing (AOR: 1.98, 95 % CI: 1.60-2.46), drug dealing (AOR: 1.59, 95 % CI: 1.30-1.94), recent incarceration (AOR: 1.47, 95 % CI: 1.09-1.98), noticing police presence (AOR: 1.30, 95 % CI: 1.06-1.60), and younger age (AOR: 1.03, 95 % CI: 1.01-1.04).

**Table 2:** Bivariable and multivariable GEE analyses of factors associated with public crack smoking in the previous six months among VIDUS and ACCESS participants who smoked crack in Vancouver, Canada (*n* = 1085)

	Unadjusted		Adjusted	
Characteristic	Odds ratio (95 % CI)	*p-*value	Odds ratio (95 % CI)	*p-*value
Age				
(per one-year decrease)	1.06 (1.05 – 1.08)	<0.001	1.03 (1.01 – 1.04)	<0.001
Gender				
(female vs. male)	1.09 (0.86 – 1.39)	0.465		
HIV serostatus				
(positive vs. negative)	0.71 (0.56 – 0.91)	0.006		
Homelessness^a^				
(yes vs. no)	4.55 (3.71 – 5.59)	<0.001	3.48 (2.77 – 4.36)	<0.001
Drug dealing^a^				
(yes vs. no)	2.76 (2.30 – 3.32)	<0.001	1.59 (1.30 – 1.94)	<0.001
Sex work^a^				
(yes vs. no)	1.94 (1.48 – 2.54)	<0.001		
Crack smoking^a^				
(≥ daily vs. < daily)	3.11 (2.59 – 3.74)	<0.001	2.69 (2.19 – 3.31)	<0.001
Shared crack pipe^a^				
(yes vs. no)	2.78 (2.31 – 3.34)	<0.001	1.98 (1.60 – 2.46)	<0.001
Binge non-injection drug use^a^				
(yes vs. no)	1.44 (1.24 – 1.67)	<0.001		
Heroin injection^a^				
(≥ daily vs. < daily)	2.21 (1.72 – 2.84)	<0.001		
Cocaine injection^a^				
(≥ daily vs. < daily)	1.48 (1.09 – 1.99)	0.011		
Crystal meth injection^a^				
(≥ daily vs. < daily)	0.89 (0.57 – 1.40)	0.626		
Public injection drug use^a^				
(Always or usually vs. sometimes, occasionally or never)	7.46 (5.42 – 10.28)	<0.001	5.42 (3.76 – 7.82)	<0.001
Noticed police presence^a^				
(yes vs. no)	1.67 (1.42 – 1.97)	<0.001	1.30 (1.06 – 1.60)	0.013
Stopped, searched or detained without arrest by police^a^				
(yes vs. no)	1.98 (1.61 – 2.44)	<0.001		
Incarceration^a^				
(yes vs. no)	2.36 (1.79 – 3.11)	<0.001	1.47 (1.09 – 1.98)	0.011
Drug addiction treatment (excluding methadone)^a^				
(yes vs. no)	0.95 (0.78 – 1.16)	0.622		
Victim of violence^a^				
(yes vs. no)	1.52 (1.22 – 1.88)	<0.001		

^a^Denotes activities/events in the previous six months

Of the 751 participants who smoked crack in public at least once, rushed public crack smoking was reported by 216 (28.8 %) participants at least once during the study period. Table 3 shows the factors positively and independently associated with rushed public crack smoking in multivariable GEE analyses, which included: homelessness (AOR: 2.61, 95 % CI: 1.96-3.49), at least daily crack use (AOR: 1.48, 95 % CI: 1.11-1.98), crack pipe sharing (AOR: 1.44, 95 % CI: 1.10-1.89), drug dealing (AOR: 1.39, 95 % CI: 1.04-1.86), and younger age (AOR: 1.02, 95 % CI: 1.01-1.04).

**Table 3:** Bivariable and multivariable GEE analyses of factors associated with rushed public crack smoking in the previous six months among VIDUS and ACCESS participants in Vancouver, Canada who smoked crack in public at least once (*n* = 751)

	Unadjusted		Adjusted	
Characteristic	Odds ratio (95 % CI)	*p-*value	Odds ratio (95 % CI)	*p-*value
Age				
(per one-year decrease)	1.04 (1.02 – 1.06)	<0.001	1.02 (1.01 – 1.04)	0.019
Gender				
(female vs. male)	1.35 (1.01 – 1.80)	0.045	1.23 (0.90 – 1.69)	0.185
Homelessness^a^				
(yes vs. no)	2.86 (2.18 – 3.76)	<0.001	2.61 (1.96 – 3.49)	<0.001
Drug dealing^a^				
(yes vs. no)	1.86 (1.41 – 2.44)	<0.001	1.39 (1.04 – 1.86)	0.026
Sex work^a^				
(yes vs. no)	1.25 (0.86 – 1.81)	0.241		
Crack smoking^a^				
(≥ daily vs. < daily)	1.71 (1.30 – 2.24)	<0.001	1.48 (1.11 – 1.98)	0.007
Shared crack pipe^a^				
(yes vs. no)	1.65 (1.28 – 2.14)	<0.001	1.44 (1.10 – 1.89)	0.008
Binge non-injection drug use^a^				
(yes vs. no)	1.28 (1.00 – 1.64)	0.051		
Noticed police presence^a^				
(yes vs. no)	1.19 (0.88 – 1.60)	0.250		
Stopped, searched or detained without arrest by police^a^				
(yes vs. no)	1.68 (1.22 – 2.31)	0.001		
Incarceration^a^				
(yes vs. no)	1.90 (1.30 – 2.78)	<0.001	1.43 (0.95 – 2.15)	0.084
Drug addiction treatment (excluding methadone)^a^				
(yes vs. no)	0.97 (0.73 – 1.28)	0.828		
Victim of violence^a^				
(yes vs. no)	1.36 (0.96 – 1.92)	0.088		

^a^Denotes activities/events in the previous six months

## Discussion

In this study, more than one-third of participants who had smoked crack reported “always or usually” smoking crack in public at some point during the study period. Of the participants who had smoked crack in public at least once, 28.8 % reported rushed public crack smoking.

This study found that individuals who were homeless had elevated odds of public crack use and rushed public crack use compared to individuals who were not homeless, which is consistent with other literature that has found that homelessness is one of the strongest predictors of using injection drugs in public [22, 33–35]. Among individuals who use crack in particular, rates of homelessness have been found to be disproportionately higher than individuals who use other illicit drugs [2]. Therefore, one way to reduce risky public crack use may involve addressing barriers to securing stable housing among this population, such as increasing accessibility to supportive housing and housing support staff who may assist with issues such as social assistance and tenancy rights [22].

In this study, individuals who smoked crack in public had a five-fold increased odds of also having injected drugs in public, which is concerning given the added risk of infection and disease transmission associated with public injection drug use [21–23]. Daily crack use and crack pipe sharing were also significantly associated with public crack use and rushed public crack use. These findings suggest that individuals who smoke crack in public may at increased risk of transmitting HIV, HCV and other infectious pathogens [7–15]. The finding that rushed public crack use was significantly associated with crack pipe sharing is especially concerning, given that rushed drug use is often characterized by inattention to hygiene and harm reduction practices, which may further exacerbate the risk of infectious disease transmission [24–26]. Therefore, greater efforts are needed to reduce barriers to distributing sterile crack pipes to reduce risky pipe sharing, especially given literature suggesting that crack pipe sharing is significantly associated with difficulty obtaining new pipes [31, 36].

Individuals who used crack in public in the present study demonstrated higher odds of recent incarceration and noticing police presence. These findings echo other research that has found that individuals who use drugs in public are often involved in the criminal justice system [23, 33, 34, 37] and are often fearful of, hassled, or shamed by police, which may perpetuate drug use in riskier environments perceived to be less exposed to police (e.g., alleys, dumpsters, hidden doorways) [26, 35]. However, such hidden environments often pose high risk for fatal overdose due to individuals being out of sight from passersby, injury or infection due to hazardous litter that is frequently found in such areas (e.g., used needles or crack pipes), or lack of access to sterile supplies in such settings [21, 26].

Individuals who reported public crack use and rushed public crack use were also more likely to have recently engaged in drug dealing. A limited body of literature has found that public crack use is associated with drug dealing [37], and that crack users are more likely than non-crack users to report illegal activities such as drug dealing as a method of income generation [15]. One potential explanation is that drug dealing and public crack use both occur at street level, where individuals may spend a large portion of their time [18]. Therefore, street-level harm reduction interventions such as utilizing street outreach programs to distribute sterile drug use equipment and provide safer drug use education may be beneficial for this population [38, 39]. Additionally, improving access to low-threshold employment opportunities for this population may reduce the need for individuals to rely on street-level income-generating activities, and in turn potentially reduce drug use and related harms [40].

Collectively, these findings suggest the potential for evidence-based public health interventions to reduce the harms associated with public crack use and rushed public crack use. While the evidence in support of supervised injection facilities is now well-established [41–45], the implementation of supervised inhalation rooms has been slow to follow suit, despite the overwhelming benefit that such facilities may offer in terms of providing a safe, non-rushed environment; sterile equipment to prevent disease transmission; direct access to health and social services for this hard-to-engage population prone to fatal overdose, infection, and other complex health concerns; immediate support for addiction treatment and counselling; reducing public drug use and related disorder; and affording considerable cost savings for health care systems [15, 46–49]. Notably, the implementation of supervised inhalation rooms need not be separate from supervised injection facilities; in fact, the delivery of both supervised injection and inhalation (or other non-injection drug use) in a single ‘safe drug use facility’ may be a more feasible and accessible approach for this population. Although individuals who use crack have previously reported high rates of willingness to use supervised inhalation rooms [27, 37, 50], policy barriers continue to impede the implementation of these potentially life-saving facilities [51].

In addition to the social and structural interventions described above, more immediate public health interventions to reduce the harms associated with public crack use may include distributing sterile crack smoking equipment and installing safe disposal containers for used crack equipment in public places [21, 35, 52, 53]. Notably, these interventions hold benefit for users of other non-injection drugs (e.g., methamphetamine, heroin), and may even play a crucial role in supporting the transition from injection drug use to less risky forms of non-injection drug use [46, 52, 54]. Furthermore, more research is needed on effective treatments for stimulant dependence, as currently no single approach has yet shown consistent evidence for effectively reducing or sustaining abstinence for stimulant use [55–59].

This study has several limitations. First, our study relied on self-reported data that is susceptible to socially desirable reporting and recall bias. Second, because the study sample was not randomly selected, these results may not be generalizable to other populations. Related to this, as there is a lack of research on crack cocaine use in general population samples—including estimates of co-occurring crack smoking and injection drug use and estimates of public crack smoking—it is difficult to compare these findings to other populations. Therefore, more research on crack smoking in general population samples is needed. Finally, as in all observational studies of this kind, the associations between the explanatory variables and the outcomes assessed may have been under the influence of unmeasured confounding, although we sought to address this bias with multivariable adjustment involving key potential correlates of public crack smoking.

## Conclusions

In summary, a high prevalence of public crack smoking and rushed public crack smoking was observed in this study. These behaviours were associated with an array of factors highlighting the vulnerability of this population, including homelessness, younger age, public injection drug use, daily crack use, crack pipe sharing, exposure to law enforcement, and involvement in drug dealing. These findings point to the need for implementing and evaluating evidence-based social, structural, and public health interventions, such as supervised inhalation facilities, to reduce the risks and harms associated with smoking crack in public.

## Abbreviations

ACCESS, AIDS Care Cohort to Evaluate Access to Survival Services; AIDS, acquired immune deficiency syndrome; AOR, adjusted odds ratio; CI, confidence interval; GEE, generalized estimating equations; HCV, hepatitis C virus; HIV, human immunodeficiency virus; QIC, independence model criterion; VIDUS, Vancouver Injection Drug Users Study
